# Highly Accurate Adaptive Federated Forests Based on Resistance to Adversarial Attacks in Wireless Traffic Prediction

**DOI:** 10.3390/s25051590

**Published:** 2025-03-05

**Authors:** Lingyao Wang, Chenyue Pan, Haitao Zhao, Mingyi Ji, Xinren Wang, Junchen Yuan, Miao Liu, Donglai Jiao

**Affiliations:** 1 College of Internet of Things, Nanjing University of Posts and Telecommunications, Nanjing 210003, China; b22100201@njupt.edu.cn (L.W.); b23100226@njupt.edu.cn (J.Y.); jiaodonglai@njupt.edu.cn (D.J.); 2 College of Communication and Information Engineering, Nanjing University of Posts and Telecommunications, Nanjing 210003, China; 1222014413@njupt.edu.cn (C.P.); jimy_email@163.com (M.J.); liumiao@njupt.edu.cn (M.L.); 3 Portland Institute, Nanjing University of Posts and Telecommunications, Nanjing 210003, China; p22000506@njupt.edu.cn

**Keywords:** adversarial attack, adversarial training, federated forest, robustness, traffic prediction

## Abstract

Current 5G communication services have limitations, prompting the development of the Beyond 5G (B5G) network. B5G aims to extend the scope of communication to encompass land, sea, air, and space while enhancing communication intelligence and evolving into an omnipresent converged information network. This expansion demands higher standards for communication rates and intelligent processing across multiple devices. Furthermore, traffic prediction is crucial for the intelligent and efficient planning and management of communication networks, optimizing resource allocation, and enhancing network performance and communication speeds and is an important part of B5G’s performance. Federated learning addresses privacy and transmission cost issues in model training, making it widely applicable in traffic prediction. However, traditional federated learning models are susceptible to adversarial attacks that can compromise model outcomes. To safeguard traffic prediction from such attacks and ensure the reliability of the prediction system, this paper introduces the Adaptive Threshold Modified Federated Forest (ATMFF). ATMFF employs adaptive threshold modification, utilizing a confusion matrix rate-based screening-weighted aggregation of weak classifiers to adjust the decision threshold. This approach enhances the accuracy of recognizing adversarial samples, thereby ensuring the reliability of the traffic prediction model. Our experiments, based on real 5G traffic data, demonstrate that ATMFF’s adversarial sample recognition accuracy surpasses that of traditional multiboost models and models without adaptive threshold modified. This improvement bolsters the security and reliability of intelligent traffic classification services.

## 1. Introduction

As fifth-generation (5G) cellular networks evolve and expand, they face several challenges, including data rates that fail to meet the required magnitude, a lack of a robust framework for cooperative transmission across multi-domain networks, the need for network innovation, and heightened security convergence demands [1]. These issues have spurred the development of Beyond 5G (B5G) [2]. B5G aims to build upon 5G by further increasing communication speeds and enhancing communication intelligence. It plans to utilize higher frequency bands for signaling, with a goal of achieving terabit-per-second data rates. Consequently, communication resources like network bandwidth and transmission delay for wireless communication are becoming increasingly valuable. The network infrastructure is under pressure to manage traffic from diverse devices, ensure load balancing, and maintain the reliability of traffic management [3,4]. Simultaneously, the intelligent development of B5G networks is emphasizing the importance of AI-based management. Cellular traffic prediction, which forecasts communication demands at future time points by learning from past data, is crucial for optimizing this management. Traffic prediction in wireless communication is crucial for optimizing resources, enhancing user experience, and planning network infrastructure. It also significantly bolsters network security. By leveraging traffic prediction, potential network attacks can be swiftly detected and mitigated, security policies can be dynamically adjusted for maximum effectiveness, and user privacy can be robustly safeguarded. These measures collectively enhance the overall security and reliability of wireless networks.

While centralized deep learning has made significant strides in addressing the accuracy and real-time requirements of traffic prediction, its predictive performance is fundamentally limited by the volume and diversity of available observed data. In the context of 5G networks, traffic data are generated by a multitude of operators and an ever-expanding array of connected devices, resulting in an exponential increase in data volume. This rapid growth not only escalates storage and communication costs but also introduces significant privacy and security risks, as sensitive data must be transmitted and stored centrally. Consequently, these challenges raise critical scalability concerns for traditional centralized models, which struggle to efficiently process and analyze such vast and distributed datasets while maintaining robust privacy protections.

To address the aforementioned challenges, federated learning has emerged as a promising solution within the realm of distributed machine learning [5]. This approach ensures that sensitive data remain within their local domain by facilitating the training of local models on client devices. Servers then aggregate these local updates to refine the global model. This not only maintains the model’s accuracy but also safeguards data privacy, significantly reducing the strain on communication costs.

While federated learning has emerged as an increasingly attractive technique for cellular traffic prediction due to its ability to leverage decentralized data while preserving privacy, it is not immune to significant security challenges. Among these, adversarial attacks [6] represent a particularly critical threat, affecting both traditional machine learning and federated learning models. These attacks involve the introduction of subtle, carefully crafted perturbations to input data perturbations that are often imperceptible to the human eye but can drastically alter the model’s output, leading to erroneous predictions. In the context of traffic prediction models, such adversarial manipulations can have severe consequences. Malicious actors could exploit these vulnerabilities to gain unauthorized control over network traffic resources, thereby disrupting server resource allocation and undermining the optimization of communication management systems. This not only compromises the reliability and efficiency of network operations but also poses risks to the overall stability and security of the communication infrastructure. Addressing these adversarial threats is therefore essential to ensuring the robustness and trustworthiness of federated learning-based traffic prediction systems.

To counter the threats of adversarial attacks on federated learning, this paper proposes the integration of a novel sample identification mechanism. This enhancement ensures the reliability of traffic prediction and intelligent network management by accurately identifying and flagging data with potential security risks. The federated forest model, as introduced by Liu in [7], offers a comparable level of model enhancement for sites with misclassification issues and delivers high-precision model optimization for those with poor or uneven data quality. This is attributable to the federated forest’s adaptability, allowing for weight adjustments to bolster model efficacy across various sites. Moreover, the federated forest model addresses communication costs by minimizing the number of model iterations, aligning with B5G’s future demands for efficiency improvements. In this study, multiple decision trees are constructed within the federated forest framework, with their prediction outcomes aggregated through voting or averaging. This approach enhances the model’s generalization capabilities by prioritizing data characteristics for classification and demonstrates robustness against noise in adversarial samples. To further refine the federated forest’s accuracy, this paper introduces an advanced model, ATMFF. ATMFF leverages confusion matrices to boost model accuracy and incorporates adaptive modification parameters within model discrimination. Notably, our approach does not impose additional communication overhead, making it a viable solution for enhancing federated learning’s resilience against adversarial attacks without compromising on efficiency.

This paper presents the following contributions:

- It examines the threats of adversarial attacks on federated learning traffic regression prediction models and empirically assesses their impact on the accuracy of real 5G cellular traffic prediction outcomes. These attacks can significantly compromise the reliability of subsequent network planning and optimization processes.
- In response to the challenges posed by adversarial attacks, this paper sets out to enhance the accuracy of traffic prediction models by focusing on the detection of adversarial samples. It addresses the uneven distribution of traffic data and the need for robustness against noise by proposing the application of federated forest (FF) to cellular traffic, aiming to achieve high recognition accuracy for adversarial samples.
- To achieve effective recognition of adversarial samples within the FF, this paper introduces a novel approach that utilizes the confusion matrix to weight the aggregation of client-local decision trees and employs an adaptive predicted results to adjust the model’s decision threshold. This method is designed to improve recognition accuracy across varying client data, thereby ensuring the high reliability of the traffic prediction model.
- This paper validates the accuracy of the proposed ATMFF in traffic identification using real 5G cellular traffic data. The results demonstrate that ATMFF outperforms traditional multiboost models and models that do not incorporate adaptive threshold modification, indicating a superior accuracy in traffic identification tasks.

## 2. Related Work

### 2.1. Federated Learning and Federated Forests

Federated learning, as introduced by McMahan [8], is a collaborative training method that enables multiple devices to co-train across different localities. This approach ensures data privacy and security by keeping sensitive information within its local domain, thereby preventing the need to transmit large volumes of sample data and reducing communication overhead. In this framework, each local base station acts as a client. Each client in the federated learning system utilizes an initial model provided by a central server for local training. Deviating from traditional machine learning, clients only share the model parameters, rather than raw data, with the central server. The server then aggregates these parameters to perform updates [9]. Federated learning has been successfully applied to a variety of machine learning tasks, including image classification [10,11], regression prediction [12,13], and time series forecasting [14,15].

In this study, we concentrate on utilizing federated learning for time series traffic prediction. Traffic prediction aims to forecast traffic usage at future time points while maintaining a high level of privacy and ensuring prediction accuracy. Current applications of federated learning in time series include the prediction of aircraft components’ remaining useful life with functional multiple perceptrons [16] and addressing data heterogeneity in spatio-temporal heterogeneous federated learning through multi-view orthogonality training [17].

Federated forest, a federated learning approach, aims to enhance accuracy by integrating multiple weak classifiers into a single, more powerful model. Liu in [7] implements a federated forest algorithm, where the central server records the optimal segmentation parameters of the decision tree based on the cardinal values in order to aggregate the models. However, this method is limited to vertically partitioned datasets. Ref. [9] optimizes feature selection and branch reduction to boost accuracy, yet it remains incompatible with horizontally segmented datasets. Ref. [18] introduces a federated forest model tailored for horizontally segmented data, addressing data heterogeneity challenges and applying it to medical care. Despite this, the model’s aggregation process requires refinement due to issues with instability and large parameter sizes. Ref. [19] proposes a novel aggregation and weight calculation method for boosting-based federated random forests, but accuracy remains suboptimal.

### 2.2. Security Threats to Federated Learning from Adversarial Attacks

Adversarial attacks involve the deliberate creation of adversarial samples designed to deceive machine learning models. Even sophisticated models, such as state-of-the-art neural networks, are susceptible to these deceptive inputs. By introducing imperceptible perturbations to original samples, these attacks can cause trained models to fail, undermining their reliability at a low cost. Notable adversarial attacks include the following: The Fast Gradient Sign Method (FGSM) attack [20] aligns perturbations with the gradient direction to maximize the loss function’s value change, thereby altering the classifier’s output. The DeepFool attack [21] employs iterative computations to gradually push samples across classification boundaries until they are misclassified. The Jacobian-based Saliency Map Attack (JSMA) [22] utilizes the Jacobian matrix of the scoring function to manipulate sample values. Finally, the Basic Iterative Method (BIM) [23] proposes a multi-step iterative process to generate adversarial samples, building upon the FGSM algorithm. These attacks can disrupt traffic prediction models with minimal noise, impacting traffic resource allocation and diminishing the dependability of network management and optimization systems.

### 2.3. Adversarial Training

To address the adversarial attacks discussed and enhance the precision of traffic prediction, the current countermeasures include the following:The Multi Steepest Descent method introduced in [24] integrates various learning strategies to enhance overall robustness. Ref. [25] emphasizes robustness measurement schemes for machine learning models, assessing their robustness via perturbation techniques. While these approaches aim to bolster model defenses, they may also increase the computational complexity and prediction time. Consequently, this could potentially diminish model accuracy and weaken their generalization capabilities.

Adversarial training represents a more prevalent strategy in combating adversarial attacks. The core concept of adversarial training is to identify adversarial samples during the testing phase by training deep neural networks to act as binary classifiers. These classifiers differentiate between clean input samples and adversarial samples. Ref. [26] differentiates adversarial from clean datasets by incorporating an outlier class into the deep learning model, utilizing maximum mean discrepancy and energy distance for this distinction. Ref. [27] suggests employing probabilistic scatter as a detector to enhance the accuracy of identifying adversarial samples. Ref. [28] trains a PixelCNN neural network to classify adversarial and clean samples based on their distinct distributions.

In order to achieve good classification results, this paper focuses on the federated forest model; the main focus of this paper will be to modify and compare the multiboost proposed by [29] and the MCC weighting model proposed by [19]. The former’s shortcomings are the low accuracy for the traffic classification task and the high complexity of the model. The latter is unable to cope with complex and variable traffic data and lacks generalization. Table 1 shows the comparison models used in this paper.

Therefore, the target model proposed in this paper not only ensures the excellent ability of adversarial sample recognition but also hopes to have high recognition accuracy and low model complexity.

## 3. Scenario Description and System Modeling

### 3.1. Description of Traffic Prediction Scenarios

**Figure 1:**
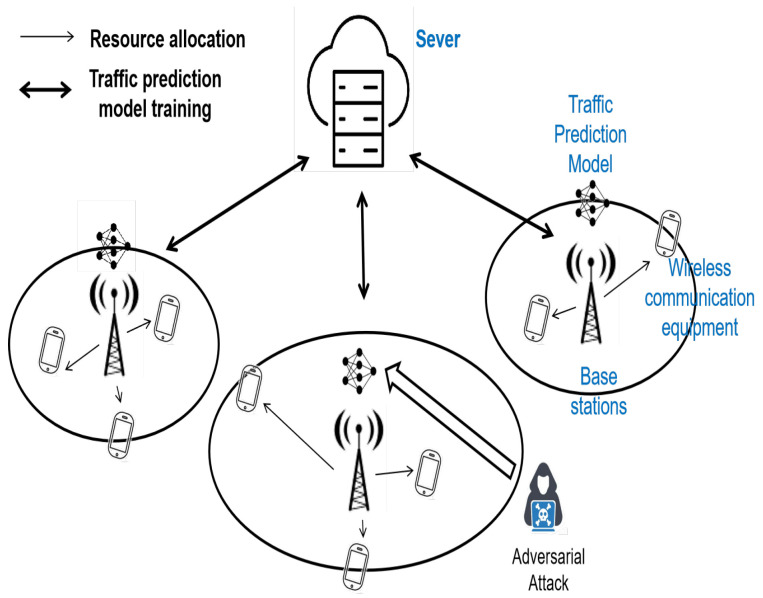
Scenarios for base station traffic prediction model training and communication resource allocation.

In a cellular traffic prediction scenario, a cloud server selects various base stations (BSs) to participate in the training of cellular traffic prediction models. Each BS preprocesses local data from mobile terminals, IoT devices, and emergency communication devices, which may include redundant information, errors, or unusual spikes. The preprocessing steps typically involve splitting the data into subsets, normalizing the data to mitigate range effects, and representing the time series data through a sliding window approach. Using the preprocessed data, the BS engages in the federated training of the model. Upon completion of the traffic prediction model training, the BS forecasts traffic usage for the next time point within its jurisdiction. Based on these predictions, the BS makes informed decisions regarding frequency resource allocation, channel resource management, scheduling, and user access control. Further details are illustrated in Figure 1.

In this paper, we focus on the security aspects of traffic prediction within cellular traffic prediction models, particularly in the context of adversarial attacks. We posit that during the traffic prediction process at the base station, there exist self-interested devices that employ the aforementioned adversarial tactics to manipulate traffic data. These actions aim to sway the traffic model’s predictions and control the model’s output, ultimately seeking to secure a greater share of communication resources.

### 3.2. Modeling of Traffic Prediction Systems

The federated learning (FL) system model depicted in Figure 1 illustrates our approach. Initially, the cloud server broadcasts the global initial mode $\omega_{0}$ to the *K* client base stations engaged in the learning process. Each base station, acting as a client in the federated learning framework, compiles its traffic data, capturing n feature points at each time interval. They aggregate data from every ten time intervals, forming a dataset. During local training, each client selects a batch size of B for the training process, yielding the model weights $\omega_{i,k}$ and model gradients $\nabla \omega_{i,k}$ for the local model, where i represents the iteration number and *k* denotes the *k*th client. Subsequently, the central server aggregates the model parameters from all clients to update the global model:

(1) $$\omega_{i+1}=\omega_{i}-\eta \times \frac{1}{N}\times \sum_{k}\omega_{i,k}$$

where $\omega_{i+1}$ denotes the global model of the i+1th round that has been updated, η is the learning rate, and *N* is the total number of clients participating in the collaborative training. In the model *F*, it is able to realize the prediction of the traffic situation at the next point in time, and the client can allocate reasonable communication resources to the device according to the prediction result.

## 4. Optimization Target

### 4.1. Description of the Problem

Given the federated learning system model and the adversarial attack scenario outlined earlier, the primary optimization objective of this paper is to develop an accurate and reliable traffic regression prediction system that can withstand the effects of adversarial attacks. A critical focus is to enhance the precision of federated learning in traffic prediction, ensuring that the model’s traffic prediction outcomes are minimally affected by adversarial samples:

(2) $$\min∥f\left(\omega ,x_{n}\right)-x_{n+1}∥$$

where *f* denotes the federated learning model for traffic prediction, and $x_{n}$ denotes the input samples, which may contain original clean samples or adversarial samples. In this paper, we need to ensure that the federated learning predicts the traffic data $f(\omega ,x_{n})$ at the next time point as much as possible to be the same as the real data $x_{n+1}$, which is our optimization objective. In this paper, we consider the idea of adversarial training, and we need to translate the above goal into recognizing as many adversarial samples as possible in the input samples:

(3) $$\max\frac{\sum_{i}F\left(x_{i}\right)==y_{i}}{\sum_{i}x_{i}}$$

Let *F* represent a binary classifier that identifies adversarial samples and assesses whether their labels match their true labels by examining the sample $x_{i}$. By maximizing the classification accuracy of the sample labels, we ensure the purity of the samples fed into the traffic prediction model *f*, thereby achieving reliable traffic prediction. Concurrently, this paper incorporates adversarial training while considering the additional computational complexity O(n), which depends on factors such as the number of samples *n*, the number of iterations, and other parameters influencing complexity.

### 4.2. Problem Transformation

This paper addresses the challenge of improving traffic prediction accuracy by focusing on the adversarial identification of input samples. In the context of federated learning for binary classification of adversarial samples, frequent data exchanges are necessary to construct the model, leading to reduced communication efficiency and increased costs. To mitigate the computational complexity constraints of the binary classification model, this paper proposes the adoption of a federated forest model for binary classification training. The federated forest, a form of ensemble learning, trains multiple weak classifiers from the initial local datasets and aggregates the classification results through weighted summation or direct voting. Its benefits include the capacity for parallel optimization on computing clusters or servers, and this parallelism can be more readily scaled to model remote datasets, as discussed in [30].

In a binary classification model, we designate the adversarial sample’s label as 1 and the original sample’s label as 0. True positive (TP) represents the count of samples that are actually labeled 1 and are also predicted as 1. False negative (FN) indicates the count of samples that are actually labeled 1 but are predicted as 0. True negative (TN) refers to the samples that are actually labeled 0 and are correctly predicted as 0. Conversely, false positive (FP) denotes the samples that are actually labeled 0 but are incorrectly predicted as 1. Consequently, the accuracy (ACC) of this binary classification model can be calculated as follows:

(4) $$ACC=\frac{TP+TN}{TP+FP+TN+FN}$$

This paper introduces a model that prioritizes the detection of adversarial samples, meaning it aims to minimize the rate at which these samples are missed (false negatives) over the rate at which original samples are incorrectly identified as antagonistic (false positives). To capture this emphasis, the recall rate is employed as a key metric in this study:

(5) $$Recall=\frac{TP}{TP+FN}$$

Hence, the optimization goal of this paper is to maximize ACC while ensuring that the recall rate is sufficiently high to prevent a significant leakage of antagonistic samples.

Additionally, we must account for the communication costs associated with training the model within a federated learning. We assess these costs in two dimensions: firstly, by the number of iterations *T* required for training the adversarial training model, and secondly, by the volume of data TI transmitted during the training process. With these considerations and optimization constraints in mind, the optimization formula for this paper can be formulated as follows:

(6) $$\begin{array}{c} \underset{\omega }{max}\left\{\;\;ACC=\frac{TP(f(\omega ,x))+TN(f(\omega ,x))}{N}\right\}\;\\ s.t.\left\{\begin{array}{c} Recall=\frac{TP(f(\omega ,x))}{TP(f(\omega ,x))+FN(f(\omega ,x))}\;\geq Recall^{*}\\ T\leq T^{*}\;\;,TI\leq TI^{*} \end{array}\right. \end{array}$$

(7) $$\begin{array}{c} TP\left(f\left(\omega ,x\right)\right)=\sum_{i}^{N}f(\omega ,x_{i})==1\&\&y_{i}==1\\ TN\left(f\left(\omega ,x\right)\right)=\sum_{i}^{N}f(\omega ,x_{i})==0\&\&y_{i}==0\\ TF\left(f\left(\omega ,x\right)\right)=\sum_{i}^{N}f(\omega ,x_{i})==0\&\&y_{i}==1\\ FP\left(f\left(\omega ,x\right)\right)=\sum_{i}^{N}f(\omega ,x_{i})==1\&\&y_{i}==0 \end{array}$$

where TP, TN, FN, and FP are the statistical results under the classification of *N* input samples, jointly determined by the federated forest model with model parameter ω and the input samples *x*, as shown in the above equations. $Recall^{*}$, $T^{*}\;$, and $TI^{*}\;$ denote the model’s target minimum recall, the maximum allowable number of iteration rounds of the model, and the maximum allowable number of bits required for model transmission. Our model is designed to achieve a reduction in the cost of communication while identifying adversarial samples as accurately as possible.

## 5. Proposed Algorithm

To fulfill the optimization objectives outlined in Section 3, this paper introduces a novel approach to evade adversarial attacks, dubbed Adaptive Threshold Modified Federated Forest (ATMFF). This approach enhances the reliability of traffic prediction by bolstering the accuracy of adversarial sample identification. In this section, we will initially detail the data preparation and other prerequisites for federated forest training. Subsequently, we will introduce ATMFF’s novel adaptive label threshold adjustment mechanism, which leverages the weighted aggregation of local decision trees based on the confusion matrix. We will also provide an analysis of its complexity.

### 5.1. Preparation of Federated Forests

In the process described above, the regression model undergoes multiple training iterations between the client and the server. Throughout this process, the client retains the gradient data $\nabla \omega_{k}^{i}$ from the learning samples, representing the gradient of the *i*th data point from the *k*th client. An adversarial attack is executed by aligning the perturbation direction with the gradient direction to maximize the change in the loss function value, thereby hindering the model’s convergence on accurate predictions. Given that the input and output dimensions of the primary task referring to [9] are not the same, each client extracts eleven features from a single time point and compiles data from ten time points to input into the model for training. However, the model’s output consists of only five features for the subsequent time point. Consequently, the adversarial attack in this paper is specifically designed to introduce interference to the sample features that correspond to the output.

(8) $${\hat{x}}_{k}^{i}=x_{k}^{i}+\varepsilon sign\left(\nabla \omega_{k}^{i}\right)$$

where sign is the symbol for obtaining the gradient, and a noise disturbance of size ε is added to the original data $x_{k}^{i}$ to obtain the adversarial sample ${\hat{x}}_{k}^{i}$. Then, labels are assigned to the original and antagonistic samples; the original label we set to 0 and the adversarial sample we set to 1. Finally, the two are randomly mixed to obtain the final sample ${\left(x,y\right)}_{adv}$.

There are multiple clients in the federated learning architecture, which are randomly selected to participate in the training of the federated forest model and are made to produce adversarial samples according to the above process. Assuming that *N* clients are given to participate in learning, their adversarial samples can be expressed as ${\left(x,y\right)}_{adv}^{i}\;\;i\in \left[1,N\right]$. where each client’s dataset is divided into a training set $S_{i}$, which is used for training the decision tree, and a test set $T_{i}$, which is used for tuning and evaluating the model.

### 5.2. Adaptive Threshold Modified Federated Forest

Traditionally, federated forest models, as referenced in [19], depend on distributional bias, data sample size, and the diversity and relevance of features during the local model training and merging process. This can lead to varying recognition effects across different clients, particularly those not part of the federated forest model training. To enhance model accuracy, adaptive adjustments are essential. Once the model aggregation is complete, the global model classifies samples based on the aggregated output. Since it is a binary classification model, each decision tree outputs either 1 or 0. If a majority of decision trees flag a sample as adversarial, the global federated forest will classify it as such. However, after outputs are weighted, the global model cannot rely solely on the median value to determine the sample label. This approach could compromise the model’s ability to make accurate judgments. To address the aforementioned issue, this paper introduces an adaptive decision threshold.

In the ATMFF framework, clients are tasked with training local forest models based on the number of decision trees specified by the server. For a given requirement of n decision trees, clients proceed to train their local models accordingly.

(9) $$f_{i}=\left[d_{1},d_{2},\ldots ,d_{n}\right]\;\;,\;\;i\in \left[1,n\right]$$

$d_{i}$ represents the decision tree within the local random forest model, and $f_{i}$ denotes the client’s local model composed of n decision trees. Once the client completes local training, the parameters of the local model are uploaded to the server. The server then aggregates these parameters to derive the global model.

After collecting the local model parameters from each client, the server integrates *f* and broadcasts the results to the participating clients, enabling the calculation of the confusion matrix for each decision tree across the various client test sets.

(10) $$f=\left[f_{1},f_{2},\ldots ,f_{N}\right]=\left[d_{1},d_{2},\ldots ,d_{N*n}\right]$$

The client computes the confusion matrix using the local test set after receiving the full model decision tree:

(11) $$c_{i}^{d}\left(d\right)=\left(TP,TN,FP,FN\right)\;,i\in \left[1,Nn\right]$$

The client uploads the traversal results to the server, which aggregates the results of each decision tree across different test sets and calculates its corresponding Matthews correlation coefficient (MCC):

(12) $$MCC=\frac{\left(TP\times TN)-(FP\times FN\right)}{\sqrt{\left(TP+FP)(TP+FN)(TN+FP)(TN+FN\right)}}$$

The MCC is used as a performance metric that shows the correlation between predicted and true values [19] in the range between [0,1]. The server filters and weights the decision tree according to its size, assuming that the MCC threshold is thred:

(13) $$\alpha_{i}^{d}=\left\{\begin{array}{c} MCC\;[i]\;MCC[i]\geq thred\\ 0\;\;\;\;\;\;MCC[i]<thred \end{array}\right.\;$$

(14) $$f^{\prime },count=\alpha_{1}^{d}\times f_{1}+\alpha_{2}^{d}\times f_{2}+\ldots +\alpha_{N*n}^{d}\times f_{N*n}$$

where $\alpha_{i}^{d}$ denotes the aggregation weight of each decision tree, at less than the threshold thred, we default it not to participate in the aggregation, and the weight size is 0. $f^{\prime }$ is the global model, and count denotes the number of decision trees of the global model.

After the federated forest completes the aggregation of weak classifiers, we can adjust the threshold adaptively based on performance metrics. Specifically, we modify the label determination threshold according to the prediction accuracy from the previous round. The accuracy rate provides a clear indication of the model’s ability to recognize adversarial samples, making it a more reliable metric than AUC, especially in the presence of data imbalance [31]. Therefore, ATMFF will primarily use the accuracy rate as the basis for threshold adjustments.

To implement adaptive threshold adjustment and enhance the federated forest’s accuracy in identifying adversarial samples, ATMFF first employs the MCC. By averaging the MCC, ATMFF establishes a preliminary threshold.

(15) $$\mathrm{BC}\_\mathrm{th}=\sum_{i}^{N*n}\alpha_{i}^{d}/count$$

During the adaptive adjustment process, we utilize the prediction results from the previous round of time nodes to enhance the classification of the adversarial model. In the calculated confusion matrix, if the probability of accurately identifying adversarial samples (AIAS) exceeds the overall accuracy, the threshold should be increased to prevent original samples from being misclassified as adversarial. Conversely, if this probability is lower than the overall accuracy, the threshold should be decreased. As the accuracy rate consistently improves and approaches 1, the threshold will eventually converge to an optimal value.

(16) $$\mathrm{BC}\_th^{\prime }=\left\{\begin{array}{c} ACC\times \sum_{i}^{N*n}\alpha_{i}^{d}/count,\;\mathrm{High}\mathrm{AIAS}\\ \frac{\sum_{i}^{N*n}\alpha_{i}^{d}/count}{ACC},\;Low\mathrm{AIAS} \end{array}\right.$$

Therefore, in the process of federated forest prediction, the model needs to adjust the binary threshold according to the prediction result of the previous round. The pseudo code is shown below.

**Figure 2:**
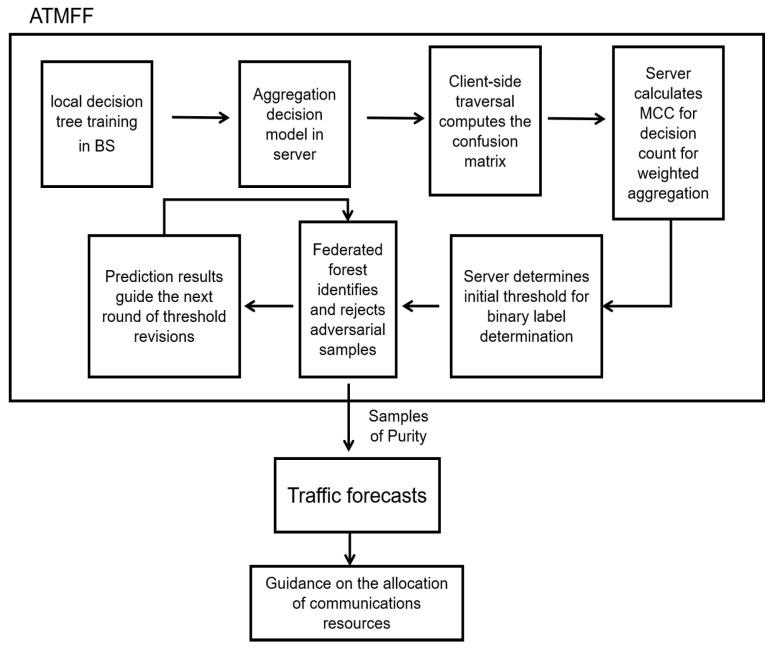
Flowchart of ATMFF-enhanced traffic prediction.

To enhance traffic prediction accuracy and ensure more reliable management of communication resources, this paper introduces the ATMFF algorithm (Algorithm 1). The final flowchart of the algorithm is presented in Figure 2.
**Algorithm 1** ATMFF1:**Input** Number of clients involved in learning *N*, Number of random forest decision trees *N*, MCC thresholds thred.2:Number of decision trees to be learned for client broadcast client;3:**for** $C^{N}\in S$ in parallel **do**4:    Training of local decision trees:5:            $f_{i}\leftarrow RandomForestClassifier\left({\left(x,y\right)}_{adv},n\right)$6:**end for**7:Server-aggregated local model8:        $f=\left[f_{1},f_{2},...,f_{N}\right]=\left[d_{1},d_{2},...,d_{N*n}\right]$9:The server sends *f* to the clients participated10:The client computes the confusion matrix for each decision tree $c_{i}^{d}\left(d\right)=\left(tp,tn,fp,fn\right)$11:The server aggregates the confusion matrix for each decision tree $\sum_{i}^{N*n}c_{i}^{d}\left(d\right)=\left(TP,TN,FN,FP\right)$12:The server computes MCC for each decision tree:13:        $MCC=\frac{\left(TP\times TN)-(FP\times FN\right)}{\sqrt{\left(TP+FP)(TP+FN)(TN+FP)(TN+FN\right)}}$14:Calculate the weighted value of the decision tree15:        $\alpha_{i}^{d}=\left\{\begin{array}{c} MCC\;[i]\;MCC[i]\geq thred\\ 0\;\;\;\;\;\;MCC[i]<thred \end{array}\right.\;$16:Aggregated global forest model17:        $f^{\prime }count=\alpha_{1}^{d}\times f_{1}+\alpha_{2}^{d}\times f_{2}+...+\alpha_{N*n}^{d}\times f_{N*n}$18:Calculate the binary classification threshold19:        $\mathrm{BC}\_th_{0}=\sum_{i}^{N*n}\alpha_{i}^{d}/count$20:Correct the threshold according to the prediction results21:**if** High accurately identifying adversarial samples **then**22:     $\mathrm{BC}\_th^{\prime }=ACC\times \sum_{i}^{N*n}\alpha_{i}^{d}/count$23:**end if**24:**if** Low accurately identifying adversarial samples **then**25:     $\mathrm{BC}\_th^{\prime }=\frac{\sum_{i}^{N*n}\alpha_{i}^{d}/count}{ACC}$26:**end if**27:Output $\mathrm{BC}\_th^{\prime }$ and $f^{\prime }$

In this study, we employ two federated models to enhance traffic prediction accuracy and reliability. The primary model is a federated regression model designed to forecast traffic data based on input data samples. Before the samples are input into the main model, they must be screened to determine if they are adversarial. To accomplish this preliminary filtering, we utilize a secondary federated model: a binary federated forest model. This model identifies whether the samples received from devices are adversarial. Original samples are then forwarded to the main task model for traffic prediction, while adversarial samples are discarded to safeguard the accuracy and reliability of the traffic predictions. A key contribution of this paper is the optimization of the federated forest model to improve the identification of adversarial samples, thereby enhancing the reliability of cellular traffic prediction. The proposed model enhances the accuracy of traffic prediction, enabling more precise resource allocation decisions. For instance, accurate forecasts of peak traffic demands allow for better frequency and channel resource allocation, minimizing congestion and improving quality of service.

### 5.3. Complexity and Communication Cost Analysis

#### 5.3.1. Calculation of Complexity

The first step of ATMFF is to build a local random model containing *k* decision trees locally, then each client uses $M_{n}$ instances to train the random forest, and the sample characteristic of each instance is *l*. Thus, the complexity of each local random forest model becomes $O(klM_{n}\log\left(M_{n}\right))$.

The second step requires the trained local model to predict and compute its confusion matrix on the test dataset, which means that $T_{n}$ test instances need to be traversed in *k* decision trees for prediction and hence the complexity of this part is $O(kT_{n})$. If the server needs to further perform the computation of confusion matrix on the test dataset of the other clients, then the result of the traversal is $O(k\sum_{i}^{N}T_{i})$.

In the last step, we need to filter each decision tree based on the results of the confusion matrix for each decision tree. First, we need to compute the overlay on the confusion matrix. Since the confusion matrix is computed by cross-computing the local models of the clients, then when there are *N* clients, there will be *N* confusion matrices for each decision tree. Each confusion needs to be superimposed to perform the superposition with a complexity of O(kN). Then, the server traverses and filters according to the updated global confusion matrix, which also has the same complexity of O(kN).

Therefore, according to the above steps, the computational complexity is $O(kfM_{n}\log\left(M_{n}\right)+k\sum_{i}^{N}T_{i}+2kN)$. And because $k,N\ll M_{n},T_{n}$, these above two complexities can also be abbreviated as $O(kfM_{n}\log\left(M_{n}\right)+k\sum_{i}^{N}T_{i})$.

In the threshold modification phase, assuming that the number of samples used for modification is *x*, this corresponds to *x* confusion matrices, and *x* results of predictions. Thus for each local model, the computational complexity of threshold modification in the training phase is o(x).

#### 5.3.2. Communication Costs

In this paper, we employ the number of bits exchanged during iterations as a metric for assessing communication costs. Initially, our model facilitates a total of three data interactions throughout the training process:

1. Local weak classifiers are exchanged between the client and the service area.
2. The client uploads the local confusion matrix.
3. The server broadcasts the global model.

Assuming that the data to be transmitted for each weak classifier is *k*, it corresponds to the need to transmit logk bits, and the total number of bits uploaded by clients in the first iteration is Nnlogk in the case where the number of weak classifiers in each local random forest is *n* and the number of clients can be N. When the server has aggregated all the local weak classifiers it needs to be broadcasted to all the clients to be used to perform the obfuscation matrix computation, which corresponds to a number of bits is $N^{2}n\;\log\;k\;$.

In the second iteration, clients upload locally trained confusion matrices, each confusion matrix contains 4s, then the amount of bit data that needs to be uploaded is Nnlog4s. after the server completes the aggregation then it needs to broadcast the global model to all clients. The number of weak classifiers after aggregation is *n*, and the amount of data transmitted is $Sn^{\prime }\times \log k$ bite when the total number of clients is *S*. The total communication cost can then be expressed as:

(17) $$TI=N\left(1+N\right)n\;\log\;k+N^{2}\mathrm{nlog}\;4s+Sn^{\prime }\times \log k$$

During the phase of threshold adjustment based on results, no extra communication costs are incurred since this process is confined to the local modification of model thresholds by the client. In contrast to traditional federated learning, which demands multiple iterations, the federated forest effectively mitigates the risk of incurring excessive interactive communication costs.

## 6. Experiment

In this section, we simulate the ATMFF to validate its accuracy in detecting adversarial samples. We utilize traffic data collected from three independent base stations in Barcelona, Spain—ElBorn, LesCorts, and PobleSec—obtained from real link control channels as referenced in [9]. These data are employed to construct a federated learning model for traffic prediction. Adversarial samples are crafted using the adversarial attacks previously discussed in Section 4. Concurrently, at each client, we generate adversarial samples from 2400 data points. During the ATMFF training phase, 4800 entries per client are utilized, totaling 14,400 samples. Of these, five-sixths are allocated to the training dataset, and one-sixth is reserved for the test dataset.

For the federated learning model, the training process consists of 23 global iteration rounds, with each global round comprising 3 local iteration rounds. The model utilizes a batch size of 128 and a learning rate of 0.001, which were determined through hyperparameter tuning to balance convergence speed and stability. We employ a Long Short-Term Memory (LSTM) network as the base architecture due to its effectiveness in capturing sequential dependencies, and the Adam optimizer is used for training to benefit from its adaptive learning rate properties.

For the federated forest model, the training process involves 100 decision trees, with each tree having a maximum depth of 10 and a minimum of 10 samples required to split internal nodes. These parameters were chosen to prevent overfitting while maintaining model interpretability and performance. During the training of the global federated forests, the parameter exchange between the server and clients is limited to a maximum of three times to minimize communication overhead and enhance scalability.

ATMFF, which primarily consists of two major modules, innovatively incorporates the use of confusion matrices for weighted aggregation of decision trees and proposes an adaptive adjustment of judgment thresholds for accurate sample classification within the federated forest. The experiments detailed in this paper include the following:

1. The accuracy of the federated forests’ weighted aggregation is enhanced through the use of mixing matrices to calculate the Matthews correlation coefficient (MCC) for weighted aggregation, as implemented in ATMFF, compared to the traditional approach of federated forest aggregation.
2. ATMFF demonstrates superior accuracy compared to federated forests by employing adaptive judgment threshold modification.

### 6.1. Accuracy of Confusion Matrix Weighted Federated Forests

First, we will evaluate the effectiveness of recognizing adversarial samples across different aggregation methods. The subsequent figure illustrates the performance of global models created using three basic federated forest aggregation techniques: full aggregation, re-voting, and random aggregation. Full aggregation involves consolidating all local weak classifiers trained by clients without any filtering. Re-voting adapts the concept of voting on weak classifiers within the federated forest, conducting a secondary vote on the local model’s outcomes to finalize the results. Random aggregation randomly filters the weak classifiers of the model. Among these methods, the first two are characterized by larger model sizes and complex computations. Meanwhile, random aggregation is plagued by instability, leading to unreliable model outcomes.

**Figure 3:**
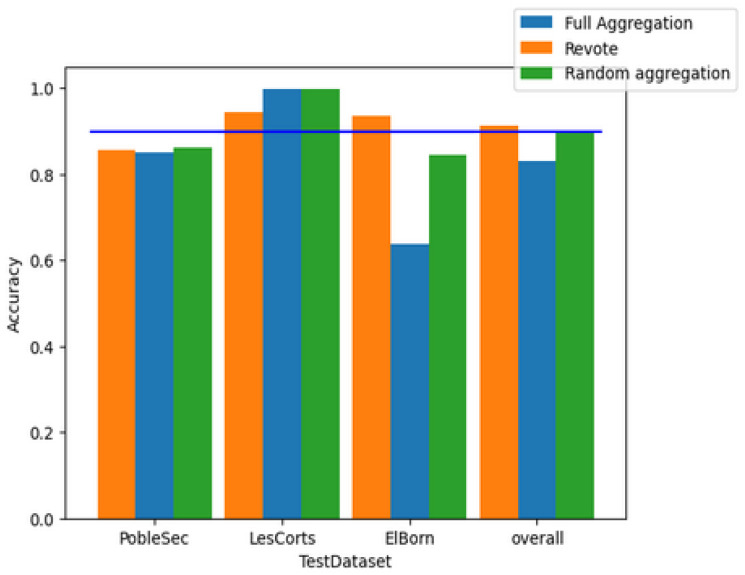
Performance of traditional aggregation.

Figure 3 reveals several issues with the outcomes of these straightforward aggregation methods: the model accuracy is suboptimal, with full aggregation achieving only 85%, and random aggregation and re-voting reaching a maximum of 90% (blue baseline). The performance of the aggregated model fluctuates significantly across different client test sets, sometimes even yielding accuracy rates lower than those of the local models.

Full aggregation is characterized by larger model sizes and complex computations. Meanwhile, multiboost [32] filters and assigns weights to weak classifiers based on their misclassification rate of test samples. We compare this approach with full aggregation. Both of them do not perform well enough in recognizing adversarial samples.

**Figure 4:**
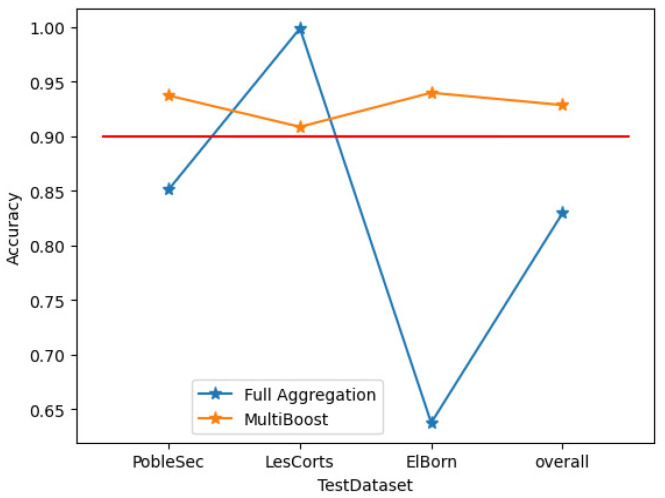
Model performance comparison between multiboost and full aggregate models.

Figure 4 reveals several issues with the outcomes of these straightforward aggregation methods: The model accuracy is suboptimal, with full aggregation achieving only 85%. The performance of the aggregated model fluctuates significantly across different client test sets, sometimes even yielding accuracy rates lower than those of the local models. However, multiboost model performance across different clients still lags behind that of locally trained models, with an overall model accuracy of 92.8%, which is only marginally superior to the aforementioned aggregation models.

This paper enhances the federated forest model by employing the MCC to refine the weighting process, thereby boosting the accuracy of adversarial sample detection. The server utilizes the MCC to sift through decision trees, assessing their performance on specific datasets. By amalgamating these performance data, the server ascertains how each decision tree performs across various test datasets, enabling a more informed filtering process. This approach is crucial for achieving enhanced global prediction outcomes.

**Figure 5:**
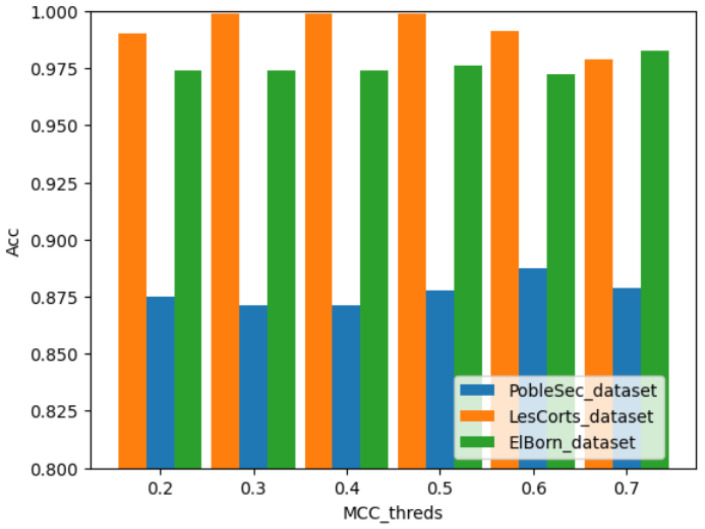
Model performance comparison between multiboost and full aggregate models.

Figure 5 illustrates the performance of the MCC-based weighted aggregation model across three clients. The horizontal axis represents the MCC threshold used for screening weak classifiers, while the vertical axis indicates model accuracy. Notably, the weighted model ensures prediction accuracy of 98% or higher for the data on clients ElBorn and LesCorts, nearly matching the accuracy of the local model on the respective local test sets. This represents an improvement of over 8% compared to the traditional aggregation method.

While the weighted model ensures high prediction accuracy for the ElBorn and LesCorts, it falls short in achieving a consistently high overall accuracy, with PobleSec’s accuracy at approximately 88%. This discrepancy may stem from the inconsistent data distribution between PobleSec and the other two clients. Additionally, the selection of the MCC threshold plays a pivotal role in determining the model’s accuracy.

**Figure 6:**
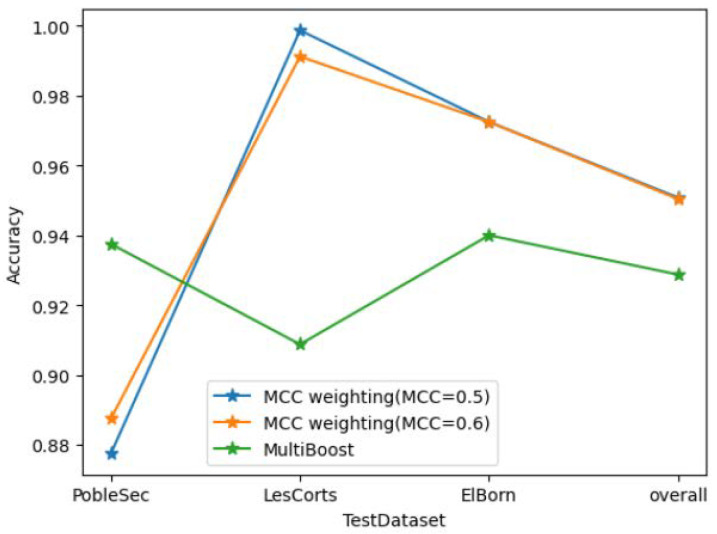
Model performance comparison between multiboost and MCC weighting models.

A higher MCC threshold results in fewer decision trees meeting the aggregation criteria. Conversely, a lower threshold leads to a greater number of aggregated decision trees. This phenomenon explains the slight dip in accuracy beyond an MCC of 0.6 in Figure 6. As the MCC threshold increases, reducing the number of decision trees, the model’s reliability diminishes, consequently lowering its accuracy.

As depicted in Figure 6, this paper directly compares the accuracy performance of using the MCC with thresholds of 0.5 and 0.6 against multiboost. Overall, the MCC weighting accuracy shows a 3% improvement. However, in the case of PobleSec, the performance is notably worse, falling below that of multiboost. Conversely, the other two clients exhibit accuracy improvements exceeding 8%.

### 6.2. Accuracy of Adaptive Threshold Modified Federated Learning

**Figure 7:**
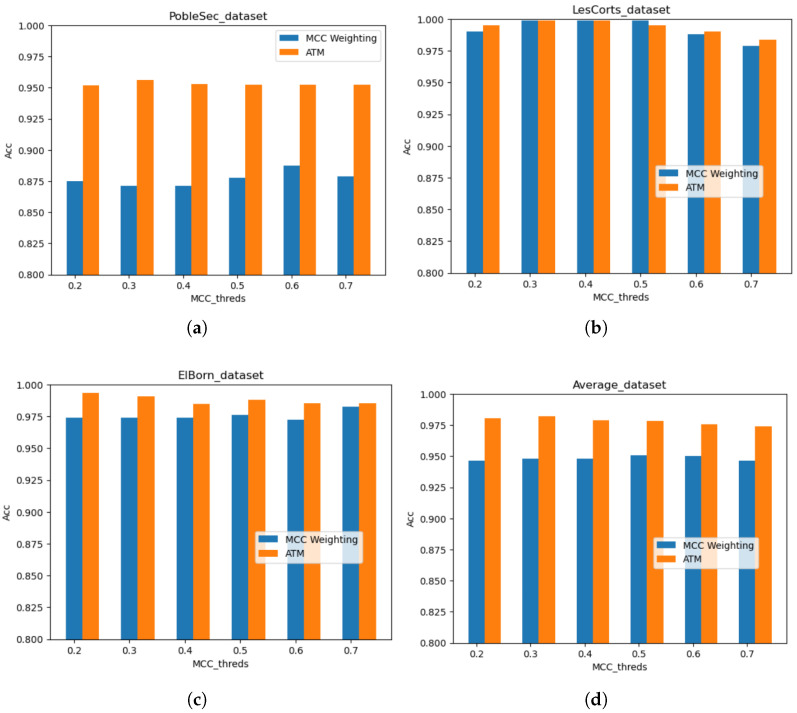
Model accuracy improvement under threshold modification. (**a**) Performance on PobleSec data. (**b**) Performance on PobleSec data. (**c**) Performance on ElBorn data. (**d**) Performance on overall data.

To enhance the model’s reliability and accuracy across various client datasets, this paper introduces a method leveraging the results of the last point in time prediction to adjust the model’s decision threshold, thereby addressing the discrepancies and distribution challenges inherent in samples from different clients. Figure 7 presents a comparison of model performances based on threshold modification. Notably, the most significant enhancement is observed in PobleSec’s test set, with accuracy increasing from 88% to 96%. Additionally, the model maintains improved accuracy in ElBorn and LesCorts. Overall, our modified model records a 3% to 4% increase in accuracy across all samples.

**Figure 8:**
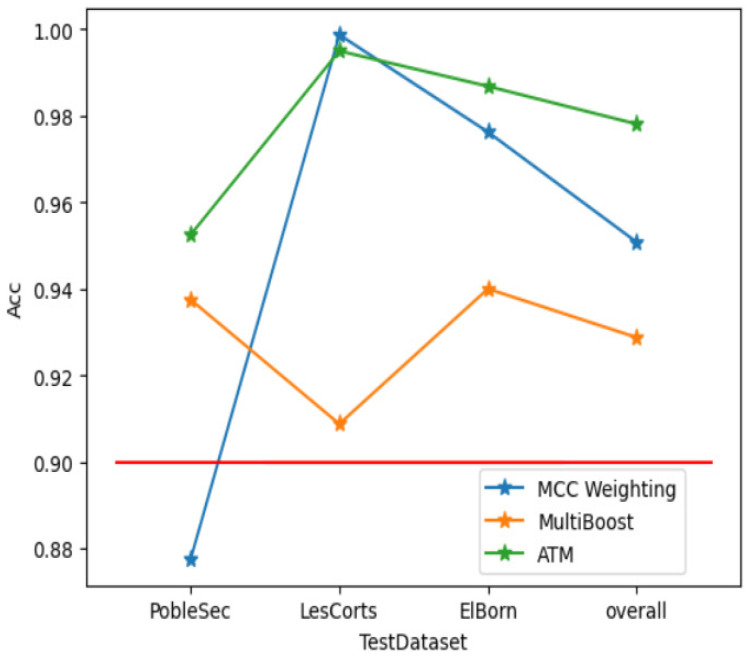
Comparative accuracy of the ATMFF against traditional aggregation methods (the x-axis shows the names of the three client base stations, PobleSec, LesCorts, and Elborn, and the overall average performance “overall”).

For instance, at an MCC value of 0.5, Figure 8 compares the outcomes of the multiboost model with ATMFF, which incorporates MCC weighting and threshold modification. This approach not only addresses the issue of uneven performance of MCC weighting across different clients but also improves accuracy by approximately 5% compared to multiboost.

Consequently, ATMFF surpasses other aggregation methods in achieving higher model accuracy, which in turn enhances the identification accuracy of adversarial samples. This ensures the reliability of the cellular traffic prediction model through the federated forest approach.

Regarding model complexity as detailed in Section 4, when the size of the model increases, although the complexity of ATMFF increases with the number of clients, the federated forests framework in ATMFF consistently offers advantages over federated learning models with the same number of clients. In terms of accuracy, our adaptive threshold adjustment mechanism enables the ATMFF model to be continuously optimized and adjusted based on the characteristics of client data and the outcomes of each prediction round. This ensures that ATMFF remains effective even in large-scale client and data scenarios.

## 7. Conclusions

This paper presents the Adaptive Threshold Modified Federated Forest (ATMFF) model, which is a novel solution designed to defend against adversarial attacks in federated traffic classification systems. In these systems, malicious actors may introduce subtle noise into input samples to deceive the model and gain more communication resources. To address this threat, we propose the ATMFF model, which integrates the principles of adversarial training and data preprocessing. This model is capable of identifying and classifying adversarial samples through an adaptive modification function that adjusts decision thresholds and a confusion matrix-based screening weighted aggregation that enhances accuracy. Moreover, ATMFF achieves efficient training with minimal iterations, thereby significantly reducing communication costs. Simulation experiments using real 5G base station traffic data from Barcelona, Spain, demonstrate that ATMFF outperforms traditional multiboost models and models without adaptive threshold modification, achieving superior classification accuracy for adversarial samples. 

## Figures and Tables

**Table 1:** Advantages and Disadvantages of Traditional Federated Forest.

	Adversarial Training	Defense Based on Data Preprocessing
Advantages	Improves model robustness	Easy to implement, no need to modify model architecture
Disadvantages	Higher cost of updating the model affects convergence speed and stability effectiveness	Depends on the feature assumptions of the input data

## Data Availability

The original contributions presented in this study are included in the article. Further inquiries can be directed to the corresponding author.
